# Comparison of Indocyanine Green Fluorescence and Technetium-99m for Sentinel Lymph Node Detection in Breast Cancer: A Retrospective Single-Center Study

**DOI:** 10.3390/diagnostics16111743

**Published:** 2026-06-05

**Authors:** Alexandra Nienhaus, Ann-Kathrin Waning, Lena Rotering, Elena Silvia Bernad

**Affiliations:** 1 Doctoral School, “Victor Babes” University of Medicine and Pharmacy, EftimieMurgu Square, No. 2, 300041 Timisoara, Romania; 2 Department of Gynecology, Klinikum Westmünsterland, 48683 Ahaus, Germany; ann-kathrin.waning@kwml.de (A.-K.W.); lena.rotering@kwml.de (L.R.); 3 Department of Obstetrics and Gynecology, Center for Laparoscopy, Laparoscopic Surgery and In Vitro Fertilization, Faculty of Medicine, Center for Neuropsychology and Behavioral Medicine, Victor Babes University of Medicine and Pharmacy, 300041 Timisoara, Romania; bernad.elena@umft.ro; 4 Ist Clinic of Obstetrics and Gynecology, Laparoscopy, In Vitro Fertilization and Embryotransfer Research Center, Pius Brinzeu County Clinical Emergency Hospital, 300723 Timisoara, Romania

**Keywords:** sentinel lymph node biopsy, indocyanine green, technetium-99m, breast cancer, fluorescence imaging, dual-tracer technique

## Abstract

**Background/Objectives:** Sentinel lymph node biopsy (SLNB) is the standard procedure for axillary staging in early breast cancer. While technetium-99m (^99m^Tc) radiocolloid is currently the standard tracer, indocyanine green (ICG) fluorescence represents a promising alternative. This study aimed to compare the detection rates of both methods in a dual-tracer setting. **Methods:** A retrospective, single-center cohort study was conducted at the Department of Gynecology, Ahaus Hospital, Germany, between April 2024 and March 2025. Fifty-two patients with malignant breast tumors underwent SLNB using both ICG fluorescence and ^99m^Tc radiocolloid. **Results:** Combined tracer use achieved a 100% sentinel lymph node detection rate. ICG alone detected nodes in 49/52 patients (94.23%), while ^99m^Tc achieved detection in 50/52 patients (96.15%). ICG identified all eight metastatic cases (100%), whereas ^99m^Tc identified seven of eight (87.5%). In obese patients (BMI ≥ 30, *n* = 10), ICG achieved 100% detection versus 90% for ^99m^Tc. The cost per application was 62.73 EUR for ICG versus approximately 250 EUR for ^99m^Tc. **Conclusions:** ICG fluorescence demonstrates comparable detection rates to ^99m^Tc with advantages in cost-effectiveness, feasibility, and performance in obese patients. ICG represents a safe, radiation-free alternative for sentinel lymph node mapping in breast cancer surgery.

## 1. Introduction

Breast cancer remains the most commonly diagnosed cancer among women worldwide [1]. The spread of breast cancer occurs via the lymphatic system to the axillary sentinel lymph nodes. In the past, radical surgery involving axillary dissection was performed. Today, however, it is well established that axillary dissection represents overtreatment due to side effects such as lymphedema, pain, seroma, and nerve injury. Consequently, sentinel lymph node biopsy (SLNB) performed in conjunction with breast tumor excision has become the standard approach for early-stage breast cancer [2,3].

The standard method for sentinel lymph node identification is currently the use of technetium-99m (^99m^Tc), which is typically injected periareolarly the day before surgery, according to the current AGO guidelines [4]. Disadvantages of this method include higher costs, limited availability of nuclear medicine specialists, radiation exposure, and the need for patients to return to the hospital the day before surgery.

A promising alternative is indocyanine green (ICG), a fluorescent dye that has been used clinically since 2005. ICG travels through the subcutaneous lymphatic flow and accumulates in the sentinel lymph node—the first draining lymph node of the breast tumor in the axilla—allowing visualization through fluorescence imaging with a specialized camera [5]. The AGO breast cancer guideline (2025) assigns a positive recommendation to indocyanine green (ICG) for sentinel lymph node mapping [4].

Although recent meta-analyses and large prospective trials, including the SENTICOL III trial and several systematic reviews from 2022–2025, have already demonstrated the non-inferiority of ICG compared with radioisotope-based mapping [6,7,8], real-world data from regional, non-academic centers remain limited. Most published evidence originates from high-volume academic institutions with dedicated nuclear medicine departments, which may not reflect the operational reality of community hospitals.

Our aim is to compare the detection rates of both methods, describe specific procedural aspects, and discuss potential complications. The added value of the present study lies in three aspects: (i) it provides real-world implementation data from a regional breast unit without on-site nuclear medicine availability, (ii) it documents the practical cost and workflow implications of replacing or supplementing ^99m^Tc with ICG, and (iii) it specifically reports performance in obese patients, a subgroup historically considered challenging for sentinel lymph node detection. A dual-tracer protocol was used to ensure patient safety during the local implementation phase of ICG and to allow head-to-head intra-patient comparison of both tracers.

## 2. Materials and Methods

A retrospective, single-center cohort study was conducted at the Department of Gynecology, Ahaus Hospital, Germany, between April 2024 and March 2025. The study included 52 patients diagnosed with malignant breast tumors who underwent sentinel lymph node (SLN) mapping using a dual-tracer technique consisting of indocyanine green (ICG) fluorescence and technetium-99m (^99m^Tc) radiocolloid.

All consecutive patients with histologically confirmed invasive breast carcinoma who underwent primary breast surgery with sentinel lymph node biopsy (SLNB) using the dual-tracer technique during the study period were screened for inclusion. Inclusion criteria were: (i) age ≥ 18 years, (ii) histologically confirmed invasive breast cancer, (iii) clinically and sonographically node-negative axilla (cN0), and (iv) availability of complete intraoperative and pathology records for both tracers. Exclusion criteria were: (i) known iodine allergy or hyperthyroidism (contraindications for ICG), (ii) prior ipsilateral axillary surgery, (iii) inflammatory breast cancer, and (iv) incomplete documentation of tracer-specific detection.

A prospective design was not feasible because ICG was being introduced into clinical routine during this period and no a priori comparative protocol was available; this is acknowledged as a limitation. No formal a priori sample size or power calculation was performed; the sample reflects the consecutive caseload of the study period, and this lack of a power calculation is explicitly acknowledged as a limitation. The type of breast surgery comprised breast-conserving surgery (*n* = 41, 78.85%) and mastectomy with primary reconstruction (*n* = 3, 5.7%) or without primary reconstruction (*n* = 6, 11.5%). Patients with who underwent neoadjuvant chemotherapy were (*n* = 16, 30%); The study was reviewed and approved by the local ethics committee of the Ärztekammer Westfalen-Lippe, and the requirement for written informed consent was waived in view of the retrospective design and use of anonymized data, in accordance with the Declaration of Helsinki.

For each patient, ^99m^Tc was administered periareolarly one day prior to surgery in accordance with standard nuclear medicine protocols. The ICG injection was performed intraoperatively, immediately following the induction of anesthesia.

For sentinel lymph node biopsy, indocyanine green (ICG) from Verdye^®^ (Diagnostic Green GmbH, Aschheim-Dornach, Germany) was used. A solution was prepared by dissolving 25 mg of ICG powder in 10 mL of sterile water for injection, resulting in a final concentration of 2.5 mg/mL. From this solution, 1 mL was injected subcutaneously or intradermally into the craniolateral quadrant of the breast scheduled for surgery, immediately prior to the procedure.

The interval between ICG injection and the initiation of sentinel lymph node excision ranged from 5 to 60 min, depending on intraoperative preparation and individual surgical conditions. Sentinel lymph node detection was performed using a gamma probe (GammaSonde, Crystal Photonics, Berlin, Germany) for the ^99m^Tc-labeled nodes, and a near-infrared fluorescence imaging system (SPY-PHI, Stryker, Kalamazoo, MI, USA) for the ICG-labeled nodes.

**Figure 1:**
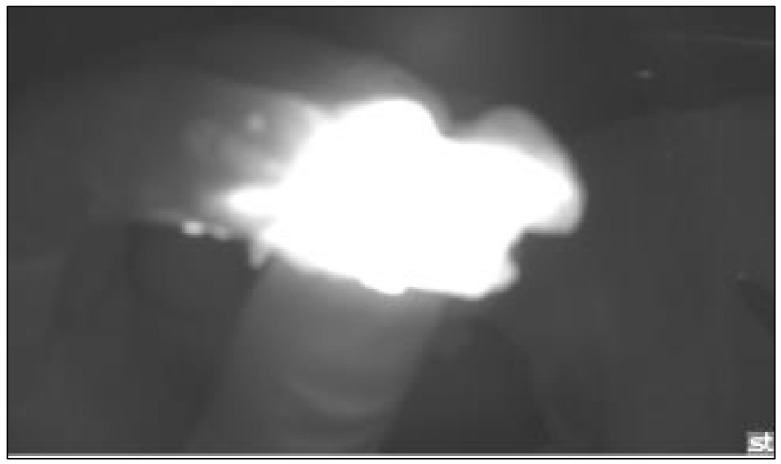
Intraoperative visualization of a sentinel lymph node using indocyanine green (ICG) fluorescence imaging.

For each patient, concordance between the two tracers was assessed intraoperatively at the nodal level: a node was considered concordant when it was both radioactive on gamma-probe counts and fluorescent under near-infrared imaging within the same anatomical position (Figure 1). Discordant nodes (radioactive only, or fluorescent only) were excised and submitted separately for pathological examination, so that the per-tracer detection rate could be calculated independently. The operating surgeon was aware of both tracer results during the procedure, as required by the dual-tracer clinical workflow; this absence of blinding is acknowledged as a potential source of detection bias and is discussed in the Limitations section.

Costs per application were calculated from institutional procurement data of the Pharmacy Department of Ahaus Hospital and included the consumable cost of the tracer dose only (one 25 mg vial of Verdye^®^ ICG, reconstitution materials, and disposable injection set for ICG; one patient dose of ^99m^Tc-nanocolloid and administration set for ^99m^Tc). The cost calculation did not include capital costs, depreciation, or maintenance of imaging equipment (gamma probe for ^99m^Tc; SPY-PHI near-infrared system for ICG), nor did it include personnel time, hospital overhead, or the additional in-patient day required for pre-operative ^99m^Tc injection. These structural cost components are addressed qualitatively in the Discussion.

### Statistical Analysis

Continuous variables are reported as mean ± standard deviation (SD) or median with range, and categorical variables as absolute and relative frequencies. Per-patient detection rates of ICG and ^99m^Tc are presented with 95% Wilson score confidence intervals (CI). Because each patient served as her own control (dual-tracer, paired design), the difference in detection rates between ICG and ^99m^Tc was assessed with McNemar’s test (with continuity correction). Detection of metastatic nodes (ICG vs. ^99m^Tc) and detection rates in the obese subgroup were compared using Fisher’s exact test, given the small number of events. A two-sided *p*-value < 0.05 was considered statistically significant. Given the limited sample size, all statistical findings are interpreted as exploratory. Analyses were performed in R version 4.3 (R Foundation for Statistical Computing, Vienna, Austria).

## 3. Results

### 3.1. Patient Cohort and Tumor Characteristics

The study cohort consisted of 52 female patients diagnosed with invasive breast cancer who underwent sentinel lymph node biopsy (SLNB) using dual-tracer mapping with indocyanine green (ICG) and technetium-99m. The mean age of the patients was 63.73 years, reflecting a predominantly postmenopausal population. The mean body mass index (BMI) was 27.25 kg/m^2^, corresponding to a slightly overweight group according to WHO classification.

Regarding histopathological tumor types, the majority of cases were invasive ductal carcinomas (44 patients, 84.62%), which is consistent with the known predominance of this subtype in breast cancer. Less frequent entities included lobular carcinoma in 1 patient (1.92%), metaplastic carcinoma in 1 patient (1.92%), invasive mucinous carcinoma in 1 patient (1.92%), mixed invasive carcinoma in 3 patients (5.77%), and tubulo-ductal carcinoma in 2 patients (3.85%).

Tumor grading revealed that the majority of tumors were moderately differentiated (G2), accounting for 78.85% (*n* = 41) of cases. Well-differentiated tumors (G1) were identified in 4 patients (7.69%), while poorly differentiated tumors (G3) occurred in 7 patients (13.46%).

Analysis of hormone receptor status demonstrated that 48 patients (92.30%) were estrogen and/or progesterone receptor positive, indicating a predominance of luminal subtypes, which are generally associated with a more favorable prognosis and responsiveness to endocrine therapy. Only 4 patients (7.70%) were receptor negative.

HER2/neu overexpression was detected in 6 patients (11.54%), which corresponds to the expected incidence of HER2 positivity in early breast cancer. The remaining 46 patients (88.46%) were HER2 negative.

Pathological examination of sentinel lymph nodes (SLNs) revealed that 44 patients (84.62%) had no evidence of lymph node metastasis (pN0 or ypN0). Micrometastatic involvement (pN1mi) was detected in 1 patient (1.92%), while macrometastatic disease (pN1a) was observed in 7 patients (13.46%). These findings are consistent with a predominantly early-stage cohort, in which the majority of patients present with limited or absent axillary involvement.

In summary, the analyzed population represents a typical cohort of early-stage, hormone receptor-positive, invasive ductal breast cancer patients, most of whom exhibit moderate tumor differentiation and no pathological lymph node involvement (Table 1). This composition provides a representative and clinically relevant foundation for assessing the diagnostic performance of dual-tracer SLNB using ICG and ^99m^Tc.

### 3.2. Number of Lymph Nodes Detected by Different Methods

Across all patients, the number of sentinel lymph nodes detected intraoperatively varied between 0 and 6 using ICG and 0 and 4 using ^99m^Tc. Pathological examination revealed a total of 183 lymph nodes, corresponding to a median of 3.51 lymph nodes per patient, confirming that multiple lymph nodes were frequently harvested and analyzed per case.

When comparing the two detection methods, at least one sentinel lymph node was successfully identified in all patients (100%) when both tracers were used in combination. The ICG technique alone demonstrated successful identification in 49 out of 52 patients (94.23%), whereas the ^99m^Tc method achieved detection in 50 out of 52 patients (96.15%).

In three patients (5.77%), ICG failed to visualize any lymph node, while ^99m^Tc was able to identify one or more nodes in all of these cases. Conversely, in two patients (3.85%), ^99m^Tc failed to detect any lymph node, although ICG fluorescence successfully localized at least one node.

In one patient (1.92%), no lymph node was identified upon histopathological examination. In this case (Patient ID 2), a sentinel lymph node was successfully detected using technetium-99m (^99m^Tc), whereas indocyanine green (ICG) failed to visualize any node. The mean number of identified sentinel lymph nodes ranged between 1 and 2 nodes per patient.

Both tracers successfully detected at least one sentinel node in 47 patients (90.38%); ICG-only detection (^99m^Tc failed) occurred in 2 patients (3.85%); ^99m^Tc-only detection (ICG failed) occurred in 3 patients (5.77%); both tracers failed in 0 patients (0%). The numbers of single-tracer failures (3 for ICG, 2 for ^99m^Tc) are therefore consistent with the per-tracer detection rates reported in the abstract (ICG 49/52 = 94.23%; ^99m^Tc 50/52 = 96.15%); the apparent inconsistency arises only when single-tracer failures are summed without reference to the paired contingency, and we have rephrased the text accordingly. Using McNemar’s test for paired proportions on the discordant pairs (2 vs. 3), the difference in detection rates between ICG and ^99m^Tc was not statistically significant (*p* = 1.00, with continuity correction). The 95% Wilson score CIs were 84.4–98.0% for ICG and 86.8–99.0% for ^99m^Tc; for the combined dual-tracer detection rate of 100%, the one-sided 95% lower confidence bound (rule of three) was 94.2%. The median number of sentinel nodes excised per patient was 2 (range 1–6) for ICG and 1 (range 0–4) for ^99m^Tc. Concordance between tracers at the nodal level was high: among the 105 sentinel nodes detected intraoperatively, 89 (84.8%) were identified by both tracers, 9 (8.6%) by ICG only, and 7 (6.7%) by ^99m^Tc only. These exploratory results indicate that ICG and ^99m^Tc provide statistically comparable per-patient detection rates in this cohort (Table 2).

### 3.3. Detection of Lymph Node Metastases

A total of eight patients with pathologically confirmed axillary lymph node metastases were identified in this study. Both indocyanine green (ICG) fluorescence and technetium-99m (^99m^Tc) radioisotope methods were compared regarding their ability to detect metastatic sentinel lymph nodes intraoperatively.

In seven of the eight cases (87.5%), both tracers successfully identified the lymph nodes that were subsequently confirmed to contain metastatic deposits on histopathological examination. Only one exception was observed—in the patient with ID 30, lymph node metastases were detected exclusively using the ICG method, while ^99m^Tc failed to identify any node in this case.

Although ICG detected at least one sentinel node in all eight metastatic cases in this cohort (8/8, 100%; 95% Wilson CI 67.6–100%), while ^99m^Tc detected at least one sentinel node in seven of these eight cases (7/8, 87.5%; 95% Wilson CI 52.9–97.8%), the small number of metastatic events (*n* = 8) does not permit a definitive conclusion regarding the superiority of either tracer for metastasis detection (Table 3). The difference between tracers was based on a single discordant case (Patient 30) and was not statistically significant (Fisher’s exact test, *p* = 1.00). These findings should be interpreted as hypothesis-generating and require validation in adequately powered cohorts.

These findings further reinforce the potential of ICG as a safe, feasible, and accurate alternative to radiotracer-based sentinel lymph node mapping, particularly in settings where nuclear medicine facilities are limited or unavailable.

### 3.4. Postoperative Complications

Postoperative complications occurred in a limited number of cases, indicating an overall favorable safety profile of the dual-tracer sentinel lymph node biopsy (SLNB) procedure. Among the 52 patients, the most frequent complication was the formation of a hematoma requiring surgical revision, observed in 7 patients (13.46%). A persistent seroma necessitating surgical revision was recorded in 1 patient (1.92%), while an axillary abscess requiring incision and drainage occurred in another 1 patient (1.92%). Similarly, one patient (1.92%) developed a postoperative wound infection, which was successfully treated with targeted antibiotic therapy.

**Figure 2:**
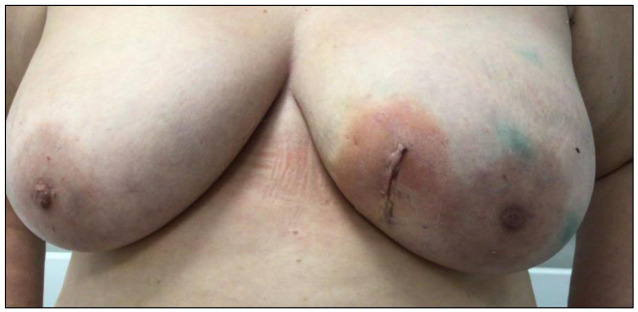
Well-known, self-limiting phenomenon of green skin color.

In addition, a temporary green discoloration of the skin at the injection site was noted in several patients following the administration of indocyanine green (ICG). This discoloration was benign and reversible, resolving spontaneously in all affected cases over time. All patients were informed preoperatively about the possibility of such a transient skin reaction as part of the standard informed consent process (Figure 2).

Importantly, no allergic reactions were reported after the use of either ICG or technetium-99m (^99m^Tc), confirming the good tolerability of both tracers. Overall, the postoperative complication rate was low, and all observed events were manageable with standard clinical interventions. These findings confirm the safety and feasibility of dual-tracer SLNB using ICG and ^99m^Tc in breast cancer surgery.

It should be acknowledged, however, that the observed rate of hematoma requiring surgical revision (7/52, 13.46%) is higher than commonly reported in the SLNB literature, where post-SLNB hematoma rates requiring re-operation are typically in the range of 1–5%. Several patient-level and procedural factors may have contributed to this rate in our cohort, including the predominance of postmenopausal patients on anti-resorptive or anti-platelet medication, the proportion of obese patients (*n* = 10, 19.23%), and the routine inclusion of breast tumor excision in the same operative session as SLNB (so that some hematomas were related to the breast wound rather than to the axillary dissection per se). The complication is not attributable to either tracer in particular. Nevertheless, this elevated rate is an internal quality signal, and a structured root-cause analysis with prospective audit of hemostasis practice has been initiated at our department (Table 4).

### 3.5. Detection Rate in Obese Patients

A subgroup analysis was performed including ten patients (19.23%) with a body mass index (BMI) ≥ 30 kg/m^2^ to evaluate the feasibility and accuracy of sentinel lymph node (SLN) detection in obese individuals. Both indocyanine green (ICG) fluorescence and technetium-99m (^99m^Tc) radiocolloid methods were compared regarding their intraoperative performance and correlation with histopathological results.

In all ten obese patients, ICG successfully identified at least one sentinel lymph node (100%), demonstrating a consistently high detection rate regardless of body habitus. The number of ICG-detected lymph nodes ranged from 1 to 6 per patient, with clear visualization of lymphatic drainage pathways. In comparison, technetium detected sentinel nodes in 9 of 10 patients (90%), failing only in one case (Patient ID 19, BMI 37 kg/m^2^) where no radioactive signal was detected, while ICG fluorescence successfully visualized the sentinel node (Table 5).

All patients except one (Patient ID 50, BMI 43 kg/m^2^) showed no evidence of lymph node metastasis (pN0) on histopathological examination. In that single case, both tracers identified the sentinel node, and metastases were confirmed (pN1a, 3/5 nodes positive).

These findings clearly indicate that ICG fluorescence imaging remains highly effective in obese patients, a population traditionally considered challenging for sentinel node detection due to increased adipose tissue thickness and reduced tissue transparency. The near-infrared light used in ICG imaging penetrates several millimeters of subcutaneous tissue, enabling reliable visualization of lymphatic channels even in individuals with elevated BMI.

In this small subgroup, ICG achieved a numerically higher detection rate than ^99m^Tc (10/10, 100% vs. 9/10, 90%), but the difference was not statistically significant (Fisher’s exact test, *p* = 1.00; 95% Wilson CIs 72.2–100% for ICG and 59.6–98.2% for ^99m^Tc). Given the very small sample size (*n* = 10), the analysis is markedly underpowered, and these findings should not be interpreted as evidence of a definite advantage of one tracer over the other. Rather, they suggest a potential advantage of ICG in obese patients, which warrants validation in larger, prospectively powered cohorts.

## 4. Discussion

Both indocyanine green (ICG) and technetium-99m (^99m^Tc) are well-established and reliable methods for the detection of axillary sentinel lymph nodes (SLNs) in patients with breast cancer. In this study, both tracers demonstrated high detection rates, confirming their diagnostic efficacy and clinical applicability in sentinel lymph node biopsy (SLNB). These results are in line with previous reports demonstrating comparable sentinel lymph node detection rates between ICG fluorescence and radioisotope methods in breast cancer patients [9,10,11].

The present study confirms that both indocyanine green (ICG) and technetium-99m (^99m^Tc) are reliable and effective tracers for sentinel lymph node (SLN) detection in patients with breast cancer. However, the ICG technique demonstrates several distinct advantages that make it particularly attractive for clinical use. It is a cost-effective, safe, and logistically simple procedure that can be performed on the day of surgery without the need for coordination with a nuclear medicine department. ICG mapping is associated with minimal adverse effects, no radiation exposure, and can be safely carried out in a standard operating room. Furthermore, the use of ICG does not require the presence of a nuclear medicine specialist, which makes the method highly accessible, especially for smaller or regional hospitals.

In contrast, the technetium-based (^99m^Tc) method presents several limitations. It requires access to nuclear medicine facilities, involves radiation exposure, and is associated with higher costs. In addition, due to its radioactive properties, patients are instructed to avoid close contact with pregnant women and children for 24 h after injection. The radiotracer must also be administered one day prior to surgery, necessitating an additional hospital visit and adding logistical complexity. These constraints can adversely affect workflow efficiency and patient comfort.

A cost comparison performed at the Department of Gynecology, Ahaus Hospital, demonstrated that the cost per application of ICG is 62.73 EUR, compared to approximately 250 EUR for ^99m^Tc. These figures reflect tracer consumable costs only (Pharmacy Department procurement data) and do not account for capital and maintenance costs of the imaging hardware. In particular, near-infrared fluorescence platforms such as the SPY-PHI system carry substantial acquisition and maintenance costs, and a comprehensive cost-effectiveness analysis would need to amortize these over the case volume of each center. Conversely, the ^99m^Tc workflow incurs structural costs not captured here, such as nuclear medicine personnel time, the additional preoperative day, and radiation safety logistics. The cost comparison should therefore be regarded as a per-procedure consumable comparison rather than a full economic evaluation, and generalizability to other healthcare systems is limited. This significant cost difference, combined with the simplified clinical workflow, underscores the economic advantage of ICG fluorescence mapping. Considering its high detection rate, safety, and financial efficiency, ICG represents a clinically and economically valuable alternative to ^99m^Tc, particularly in healthcare settings lacking nuclear medicine infrastructure.

ICG fluorescence imaging appeared to perform well in the obese subgroup of our cohort (*n* = 10), in whom increased adipose tissue may impair visualization of lymphatic pathways. In this small subgroup, ICG identified at least one sentinel node in all patients, while ^99m^Tc was successful in 9 of 10. However, given the very small sample size and the non-significant statistical comparison (Fisher’s exact test, *p* = 1.00), these findings suggest only a potential advantage of ICG in obese patients, which warrants validation in larger, prospectively designed cohorts. ICG also identified the metastatic case in this subgroup; this single observation should not be interpreted as a definitive statement on the staging performance of ICG.

The only relevant contraindications for ICG use are a known iodine allergy or hyperthyroidism, as ICG contains iodine in its molecular structure. Adverse reactions to ICG are rare but have been described, including isolated hypersensitivity responses [12]. According to the current Arbeitsgemeinschaft Gynäkologische Onkologie (AGO) guidelines, the application of ICG in sentinel lymph node mapping is still considered off-label, and therefore, patients must be informed preoperatively about this regulatory status. In addition, all patients should be counseled about the possibility of transient green skin discoloration at the injection site, which is benign and self-limiting.

Consistent with the findings of the present study, ICG has been rated with a positive (+) recommendation in the current AGO guideline [4]. Given its excellent safety profile, high detection rate, and practical advantages, it can be expected that future updates of the guideline may further strengthen this recommendation.

Two recent studies evaluating ICG as the sole tracer for sentinel lymph node biopsy reported excellent detection rates of up to 98.4%, thereby reinforcing the diagnostic reliability of this method. Pellini et al. [13] confirmed the safety and feasibility of ICG-only mapping, while Hartmann et al. [14] demonstrated high detection accuracy in early breast cancer using ICG marking of axillary sentinel lymph nodes.

Our exploratory results are consistent with the broader contemporary literature. Large meta-analyses comparing ICG with radiocolloid-based SLNB in breast cancer have consistently reported detection rates above 95% and non-inferiority of ICG in terms of SLN identification [6,7]. The SENTICOL III trial in cervical cancer [8], together with more recent multicenter evaluations in breast cancer, has further established ICG fluorescence as a clinically robust mapping technique. The present cohort therefore confirms, in a real-world regional setting, what has already been demonstrated in academic and trial-based environments.

Two methodological aspects deserve specific discussion. First, the interval between intraoperative ICG injection and sentinel lymph node excision in our protocol varied between 5 and 60 min, depending on the surgical sequence and patient setup. While ICG fluorescence has a broad practical window (the dye remains visible in lymphatic channels for several minutes to tens of minutes after injection), this variability could in principle influence detection rates and the number of nodes visualized. We did not perform a formal subgroup analysis stratified by injection-to-excision interval because of the small sample size, but we note this as a methodological limitation and an important parameter to standardize prospectively. Second, both tracer results were available to the operating surgeon during the procedure (gamma probe counts and fluorescence imaging), and patients were treated under a real-world dual-tracer workflow rather than under blinded conditions. This is intrinsic to the dual-tracer design and could inflate the apparent agreement between the two methods (verification bias); a fully blinded comparative design is ethically difficult in this context but should be considered for future prospective studies.

An important consideration for the long-term implementation of ICG-based SLNB is the question of oncological safety. Our study reports intraoperative and short-term outcomes only; it does not address axillary recurrence, disease-free survival, or false-negative rates of ICG mapping in larger cohorts. Published evidence has demonstrated false-negative rates for ICG-guided SLNB in breast cancer that are broadly comparable to those of dual-tracer techniques (typically <10% in cN0 disease) [6,7], but long-term oncological outcome data—including regional recurrence after ICG-only mapping—remain limited and constitute a clear priority for future research. Until such long-term data accumulate, particularly from large prospective registries, ICG should be implemented with appropriate clinical caution and ideally with audit of axillary recurrence rates at the institutional level.

In summary, ICG represents a safe, radiation-free, and cost-effective alternative to technetium-based mapping, providing comparable detection rates with additional practical advantages relating to logistics, cost, and a possible benefit in obese patients. With growing evidence supporting its efficacy, ICG is expected to play an increasingly important role in sentinel lymph node biopsy, and it is hoped that future AGO guideline updates will endorse this technique with a double-positive (++) recommendation.

### Limitations

Several limitations of this study warrant explicit acknowledgement. (i) The study has a retrospective design, which carries an inherent risk of selection and information bias despite the use of consecutive eligible patients. (ii) It is a single-center experience from a regional breast unit, which limits external generalizability; multicenter validation is needed. (iii) The total sample size (*n* = 52) and, in particular, the small number of metastatic events (*n* = 8) and obese patients (*n* = 10) are insufficient to detect modest between-tracer differences with adequate statistical power; no a priori sample size or power calculation was performed. (iv) The dual-tracer protocol itself can inflate combined detection rates: by definition, the combined detection rate cannot be lower than the detection rate of either single tracer, and patients in whom one tracer fails still benefit from the other. This may have favorably affected the safety and feasibility profile reported here. (v) The operating surgeon was not blinded to the results of either tracer, introducing potential detection bias. (vi) The interval between ICG injection and node excision was not strictly standardized (5–60 min), and no learning curve analysis was performed. (vii) The cost comparison is based on local consumable procurement data and does not include capital, maintenance, or personnel costs; it is not a formal cost-effectiveness analysis. (viii) Long-term oncological outcomes, including axillary recurrence and false-negative rates, were not assessed. These limitations should be considered when interpreting the results, and they directly motivate the design of larger, prospective, multicenter studies.

## 5. Conclusions

In this retrospective single-center cohort, ICG fluorescence imaging demonstrated detection rates that were statistically comparable to those of the established technetium-99m method (94.23% vs. 96.15%, McNemar *p* = 1.00). The dual-tracer approach achieved a combined per-patient detection rate of 100% (95% one-sided lower bound 94.2%). Although ICG identified all eight metastatic cases in this cohort, the small number of metastatic events does not permit a definitive conclusion regarding superior sensitivity. ICG showed consistently high detection in the obese subgroup (*n* = 10), but this finding is underpowered and requires confirmation in larger cohorts.

With its favorable safety profile, absence of radiation exposure, simplified logistics, and substantially lower consumable costs (62.73 vs. 250 EUR per application), ICG represents a viable and attractive alternative to radiotracer-based sentinel lymph node mapping, particularly in regional centers without on-site nuclear medicine. Future prospective, multicenter studies with larger patient cohorts and long-term oncological follow-up are warranted to further validate these findings.

## Figures and Tables

**Table 1:** Patient Cohort and Tumor Characteristics (*n* = 52).

Parameter	Category	Result	Percentage
Mean age		63.73 years	-
Mean BMI		27.25 kg/m^2^	-
Histological type	Invasive ductal	44	84.62%
	Lobular	1	1.92%
	Metaplastic	1	1.92%
	Invasive mucinous	1	1.92%
	Mixed invasive	3	5.77%
	Tubulo-ductal	2	3.85%
Grading	G1	4	7.69%
	G2	41	78.85%
	G3	7	13.46%
Hormone receptor	Positive	48	92.30%
	Negative	4	7.70%
HER2/neu status	Positive	6	11.54%
	Negative	46	88.46%
SLN pathology	pN0/ypN0	44	84.62%
	pN1mi	1	1.92%
	pN1a	7	13.46%

**Table 2:** Summary of sentinel lymph node detection by tracer (*n* = 52).

Parameter	ICG	^99m^Tc
Per-patient detection rate, *n*/*N* (%)	49/52 (94.23)	50/52 (96.15)
95% CI (Wilson)	84.4–98.0%	86.8–99.0%
SLNs excised per patient, median (range)	2 (0–6)	1 (0–4)
Single-tracer failures, *n* (%)	3 (5.77)	2 (3.85)
Combined dual-tracer detection rate	52/52 (100%); 95% one-sided lower bound 94.2%	
McNemar test (ICG vs. ^99m^Tc)	*p* = 1.00 (continuity correction)	

**Table 3:** Comparison of Detection Methods for Axillary Lymph Node Metastases (*n* = 8).

**Patient ID**	**ICG Nodes**	**^99m^** **Tc Nodes**	**Path. Result**	**Pos./Total**
1	1	1	pN1mi	1/1
4	1	2	pN1a	2/4
9	2	1	pN1a	2/3
17	4	3	pN1a	2/4
30	1	0	pN1a	1/2
33	1	1	pN1a	1/5
50	6	1	pN1a	3/5
51	4	4	pN1a	1/3

**Table 4:** Postoperative Complications (*n* = 52).

Complication	*n*	%
Hematoma requiring surgical revision	7	13.46%
Persistent seroma requiring surgical revision	1	1.92%
Abscess requiring incision and drainage	1	1.92%
Wound infection requiring antibiotic therapy	1	1.92%
Allergic reaction	0	0.00%

**Table 5:** Summary of sentinel lymph node detection in the obese subgroup (BMI ≥ 30 kg/m^2^, *n* = 10).

Parameter	ICG	^99m^Tc
Detection rate, *n*/*N* (%)	10/10 (100)	9/10 (90)
95% CI (Wilson)	72.2–100%	59.6–98.2%
BMI of patients, median (range), kg/m^2^	33 (31–43)	
Fisher’s exact test (ICG vs. ^99m^Tc)	*p* = 1.00	
Metastatic cases in subgroup, *n*	1 (Patient 50; pN1a; detected by both tracers)	

## Data Availability

The data presented in this study are available on request from the corresponding author. The data are not publicly available due to patient privacy restrictions.

## References

[1] Sung H., Ferlay J., Siegel R.L., Laversanne M., Soerjomataram I., Jemal A., Bray F. (2021). Global Cancer Statistics 2020: GLOBOCAN Estimates of Incidence and Mortality Worldwide for 36 Cancers in 185 Countries. CA Cancer J. Clin..

[2] Oeffinger K.C., Fontham E.T.H., Etzioni R., Herzig A., Michaelson J.S., Shih Y.C., Walter L.C., Church T.R., Flowers C.R., LaMonte S.J. (2015). Breast Cancer Screening for Women at Average Risk: 2015 Guideline Update From the American Cancer Society. JAMA.

[3] Zhang X., Li Y., Zhou Y., Mao F., Lin Y., Guan J., Sun Q. (2016). Diagnostic Performance of Indocyanine Green-Guided Sentinel Lymph Node Biopsy in Breast Cancer: A Meta-Analysis. PLoS ONE.

[4] ArbeitsgemeinschaftGynäkologischeOnkologie (AGO) (2025). Diagnostik und Therapie des Mammakarzinoms-Empfehlungen der AGO Kommission Mamma, Version 2025.1.

[5] Kitai T., Inomoto T., Miwa M., Shikayama T. (2005). Fluorescence Navigation with Indocyanine Green for Detecting Sentinel Lymph Nodes in Breast Cancer. Breast Cancer.

[6] Goonawardena J., Yong C., Law M. (2020). Use of Indocyanine Green Fluorescence Compared to Radioisotope for Sentinel Lymph Node Biopsy in Early-Stage Breast Cancer: Systematic Review and Meta-Analysis. Am. J. Surg..

[7] Liu J., Huang L., Wang N., Chen P. (2017). Indocyanine green detects sentinel lymph nodes in early breast cancer. J. Int. Med. Res..

[8] Lecuru F.R., McCormack M., Hillemanns P., Anota A., Leitao M., Mathevet P., Zweemer R., Fujiwara K., Plante M., Goffin F. (2019). SENTICOL III: An International Validation Study of Sentinel Node Biopsy in Early Cervical Cancer. A GINECO, ENGOT, GCIG and Multicenter Study. Int. J. Gynecol. Cancer.

[9] Jimbo K., Nakadaira U., Watase C., Murata T., Shiino S., Takayama S., Suto A. (2023). Clinical Significance of Discordances in Sentinel Lymph Node Reactivity between Radioisotope and Indocyanine Green Fluorescence in Patients with cN0 Breast Cancer. Asian J. Surg..

[10] Papathemelis T., Jablonski E., Scharl A., Hauzenberger T., Gerken M., Klinkhammer-Schalke M., Hipp M., Scharl S. (2018). Sentinel Lymph Node Biopsy in Breast Cancer Patients by Means of Indocyanine Green Using the Karl Storz VITOM^®^ Fluorescence Camera. Biomed. Res. Int..

[11] Staubach P., Scharl A., Ignatov A., Ortmann O., Inwald E.C., Hildebrandt T., Gerken M., Klinkhammer-Schalke M., Scharl S., Papathemelis T. (2021). Sentinel Lymph Node Detection by Means of Indocyanine Green Using the Karl Storz VITOM^®^ Fluorescence Camera: A Comparison between Primary SLNB versus SLNB after Neoadjuvant Chemotherapy. J. Cancer Res. Clin. Oncol..

[12] Garski T.R., Staller B.J., Hepner G., Banka V.S., Finney R.A. (1978). Adverse Reactions after Administration of Indocyanine Green. JAMA.

[13] Pellini F., Bertoldi L., Deguidi G., Perusi N., Caldana M., De Flaviis M., Di Paolo S., Mirandola S., Tombolan V., Zambelli Sopalu S. (2022). The Use of Indocyanine Green as the Only Tracer for the Identification of the Sentinel Lymph Node in Breast Cancer: Safety and Feasibility. Gland Surg..

[14] Hartmann S., Plonus M.L., Schultek G., Stubert J., Gerber B., Reimer T. (2024). Indocyanine Green Marking of Axillary Sentinel Lymph Nodes in Early Breast Cancer. Geburtshilfe Frauenheilkd..

